# Diagnostic Performance of Soluble Triggering Receptor Expressed on Myeloid Cells-1 in Ventilator-Associated Pneumonia of Patients with Ischemic Stroke

**DOI:** 10.1155/2017/9513690

**Published:** 2017-02-22

**Authors:** Yuetian Yu, Cheng Zhu, Chunyan Liu, Yuan Gao, Rong Yin, Jianguo Cao

**Affiliations:** ^1^Department of Critical Care Medicine, Renji Hospital, School of Medicine, Shanghai Jiao Tong University, Shanghai 200001, China; ^2^Department of Emergency Medicine, Ruijin Hospital, School of Medicine, Shanghai Jiao Tong University, Shanghai 200025, China; ^3^Department of Emergency Medicine, Minhang Central Hospital, School of Medicine, Fudan University, Shanghai 201100, China

## Abstract

*Objective*. To investigate the effect of soluble triggering receptor expressed on myeloid cells-1 (sTREM-1) in serum, bronchoalveolar lavage fluid (BALF), endotracheal aspiration (ETA), and exhaled breath condensate (EBC) samples as early biomarkers for the diagnosis of ventilator-associated pneumonia (VAP) in patients with ischemic stroke.* Methods*. One hundred and thirty-two patients with clinically suspected VAP were enrolled in this study. Bronchoscopy was performed on the day of clinically suspected VAP. sTREM-1 levels in serum, BALF, ETA, and EBC were measured. VAP was diagnosed by quantitative cultures of BALF (≥10^4^ cfu/mL).* Results*. VAP was confirmed in 76 (57.58%) cases. Patients with VAP showed significantly higher sTREM-1 in BALF [32.35 (IQR, 30.08–41.72) versus 18.92 (11.89–31.72)] pg/mL and in EBC [1.57 (IQR, 1.02–2.61) versus 0.41 (0.19–1.61)] pg/mL than patients without VAP. The area under the curve was 0.813 (*p* < 0.001). The optimum cut-off value for sTREM-1 in BALF was 23.61 pg/mL, yielding sensitivity and specificity of 85.5% and 73.1%. sTREM-1 in BALF had excellent correlation with that in EBC (*R*^2^ = 0.78, *p* < 0.05).* Conclusions*. sTREM-1 in EBC and BALF had good diagnostic performance in differentiating patients with and without VAP.

## 1. Introduction

Ventilator-associated pneumonia (VAP) which constitutes a frequent infection in intensive care unit (ICU) patients consumes vast healthcare resources and increases proportionally to the duration of ICU stay and mortality [1]. The incidence of VAP varies depending on the study population. For example, in patients with ischemic stroke who are characterized by advanced age, depressed level of consciousness, immune suppression, and long-term bed rest, the incidence of VAP can increase to approximately 40%, which is associated with a less favorable neurologic and functional outcome [2]. It is recognized that one-third to half of all VAP-related deaths are directly attributable to pneumonia. Diagnosis of VAP with clinical suspicion is overly sensitive with low specificity, leading to unnecessary antibiotics use [3], and the incidence of VAP among ICU ischemic stroke patients has not been thoroughly investigated.

The triggering receptor expressed on myeloid cells-1 (TREM-1) is a member of the immunoglobulin superfamily. Its expression on phagocytes is upregulated by exposure to bacteria and fungi. A soluble form of TREM-1 (sTREM-1) was proposed as a new biomarker which had been tested for acute infections with different diagnostic and prognostic value [4]. sTREM-1 can be found in different body fluids, such as serum, bronchoalveolar lavage fluid (BALF), endotracheal aspiration (ETA), and exhaled breath condensate (EBC), where it can be assayed by ELISA using commercial immunoassay kits [5]. Some clinical studies have proved that sTREM-1 did have the ability to identify patients with sepsis while others come to an opposite conclusion [6, 7].

However, the real effect of sTREM-1 on diagnosis of VAP is still unknown and has not been well evaluated yet. Furthermore, the majority of the studies about sTREM-1 levels in VAP were heterogeneous due to including different diseases. What is the most important is that some of these studies did not exclude the patients with extrapulmonary infection which might lead to misinterpretation of the study results. The subjects of our study were patients with ischemic stroke, who had a high incidence of VAP and were without any infectious complications at the time of invasive mechanical ventilation.

Trauma, malignant neoplasms, and acute inflammatory response could all lead to the raise of sTREM-1 in serum due to its high sensitivity and low specificity which resulted in the poor performance in VAP diagnosis. BALF was partly secreted by lung which was infected as the target organ. So we measured sTREM-1 levels in serum, BALF, ETA, and EBC samples from patients who underwent bronchoscopy for a clinical suspicion of VAP and we hypothesized that sTREM-1 in different body fluid might have different values in diagnosing VAP.

## 2. Methods

### 2.1. Research Briefs

The present study was a multicenter (155 ICU beds in total) prospective observational trial which included samples of serum, BALF, ETA, and EBC from eligible patients. The study was approved by the Review Board and Ethics Committee of Shanghai Jiaotong University School of Medicine (No: 2013-Clinical-Res-085) and informed consent was obtained for all patients, from either the patient or the next of kin. The study was performed from January 2013 to December 2015. One month before the start of this study, a standardized sampling, processing, analysis, and statistics procedure was set by 9 investigators from these centers after 3 days' learning and discussion. All of the centers carefully followed this procedure during the study time. There was no significant difference in compliance among the centers.

### 2.2. Study Population

Consecutive sampling was used to recruit critical ill patients. Patients were eligible for enrolment if they were diagnosed with ischemic stroke by magnetic resonance imaging (MRI), underwent bronchoscopy while mechanically ventilated for clinically suspected VAP, and aged between 18 and 80 years. Patients were excluded from the study if they were moribund or not expected to survive 3 days because of an underlying irreversible medical condition, had active pneumonia when admitted to ICU or had extrapulmonary infection during ICU stay, were immunocompromised, were with malignancies, and were pregnant or if informed consent could not be obtained.

### 2.3. Diagnosis, Treatment, and Prevention of VAP

VAP was suspected if the patient had a radiographic infiltrate that was new or progressive, together with clinical findings that were suggestive of infection, such as the onset of fever (temperature ≥38.3°C), leukocytosis (≥10 × 10^9^/L or ≤4 × 10^9^/L), purulent sputum, and decline in oxygenation. In addition, a positive BAL culture (≥10^4^ colony-forming units/mL, cfu/mL) was required to confirm the diagnosis of VAP [8]. The protocol for VAP treatment and prevention followed standard protocols in all institutions also based on accepted guidelines. Care bundles for preventing VAP were also employed [3, 8].

### 2.4. Clinical Assessment

Baseline assessment included the evaluation of demographic data (age and gender), medical history, stroke subtype, ratio of partial oxygen to fraction of inspired oxygen (PaO_2_/FiO_2_), acute physiology and chronic health evaluation (APACHE II), Glasgow Coma Scale (GCS), modified clinical pulmonary infection score (mCPIS), and the level of inflammatory biomarkers.

### 2.5. Samples Processing and Measurement

Bronchoscopy was performed on the day when VAP was suspected and all samples were collected for measurement at the same day. The diagnostic flexible bronchoscopy guideline of British Thoracic Society was also followed during the performance [9]. Selection of the segment for bronchoalveolar lavage (BAL) was guided by chest X-ray changes. The right middle lobe or lingual lobe was selected when diffuse infiltrates were present. Five aliquots of 20 mL of sterile saline were instilled and aspirated gently. The first aliquot was discarded and the subsequent four aliquots were pooled for analysis. Part of the first BALF specimen was sent to the laboratory immediately after collection for measurement of sTREM-1 levels; the remainder of the sample was sent to the microbiology laboratory for quantitative culture. Quantitative culture of BALF retrieved by direct bronchoscopic methods yields the best sensitivity and specificity to diagnose VAP and can differentiate true infection from colonization or inflammation. Culture-positive BALF was defined as a count ≥10^4^ cfu/mL. Thus, the diagnosis of pneumonia was confirmed by a positive BALF culture [10].

The EBC fluid was collected in a plastic container located in the center of the exhaled portion of the ventilator tubing (mid-way between the ventilator and the patient's endotracheal tube). EBC was centrifuged and cell-free supernatants were aliquoted into 2 polypropylene tubes and stored at −80°C.

ETA fluid was collected using a feeding tube, which was introduced down the endotracheal tube until resistance was felt. Then, using a syringe, 1 ml/kg of normal saline (maximum 20 ml) was instilled through the feeding tube followed by a small amount of air to clear the dead space of the endotracheal tube and suction was applied to obtain the fluid [11].

Blood samples (5 mL) from median basilic vein were separated by centrifugation (TDL-60C, 6000 revolutions per minute, China) and stored at −80°C in anticoagulative tubes.

Commercially available ELISA assays were used for detecting sTREM-1. The detection limit of the methods for sTREM-1 was 0.05~1600 pg/mL (SEA571Hu96 Tests, USA). All samples were run in duplicate. To minimize variations in samples collection, the procedure was performed by the same investigator.

### 2.6. Statistical Analysis

Statistical analysis was performed using SPSS version 19.0 (IBM for Windows). Data were initially assessed for normality and log transformed where appropriate. Data between the VAP positive and VAP negative were compared using Chi-square test for equal proportion or Fisher exact test where numbers were small with results presented as percentages (*n*). Continuously normally distributed variables were compared using Student's *t*-test and presented as means (standard deviations), whereas nonnormally distributed data was compared using Wilcoxon rank-sum test and reported as medians (interquartile range). The statistical tests performed were two-sided. All analysis was performed on an intention-to-treat basis and a two-sided *p* < 0.05 was considered to be statistically significant. Figures were drawn using Graphpad prism version 5.0 and Medcalc11.4.2.

## 3. Results

### 3.1. Characteristics of the Patients

Over the study period, 328 patients with acute ischemic stroke and requiring mechanical ventilation were admitted to ICU. Thirty-two patients were excluded because informed consent was not obtained. One hundred and sixty-four patients who had active pneumonia when admitted or had extrapulmonary infection during ICU stay were also excluded. One hundred and thirty-two patients clinically suspected of VAP were included in the study. Among them 76 (57.58%) were diagnosed of VAP while 56 were not. The baseline characteristics of the 132 patients were shown in Table 1. The demographic data, position of stroke, PaO_2_/FiO_2_, APACHE II, GCS, and modified CPIS were not significantly different between patients with or without VAP (*p* > 0.05).

### 3.2. Bacteria Detection

BALF culture was positive (considering the cut-off of >10^4^ cfu/mL) in all the 76 patients with VAP, with the growth of the following agents: Gram-negative bacterium (*n* = 62, 81.58%). The top three Gram-negative bacteria were* Pseudomonas aeruginosa* (*n* = 28),* Klebsiella pneumoniae* (*n* = 15), and* Acinetobacter baumannii* (*n* = 11). Five Methicillin-resistant* Staphylococcus aureus* were also detected in 14 strains of Gram-positive bacterium. Blood culture was positive in 4 patients with VAP, with the growth of* Staphylococcus aureus* methicillin-resistant (*n* = 3) and* Staphylococcus aureus* methicillin-sensitive (*n* = 1).

### 3.3. sTREM-1 Detection and Comparison

sTREM-1 was detected in all the 132 patients at the day of VAP suspicion. The levels of sTREM-1 in serum, BALF, ETA, and EBC between two groups were compared. BALF sTREM-1 concentrations were higher in patients with VAP than patients without VAP [32.35 (IQR, 30.08–41.72) versus 18.92 (11.89–31.72)] pg/mL as well as EBC sTREM-1 concentrations [1.57 (IQR, 1.02–2.61) versus 0.41 (0.19–1.61)] pg/mL (*p* < 0.05). sTREM-1 levels were consistently higher in BALF than EBC in all patients.

**Figure 1:**
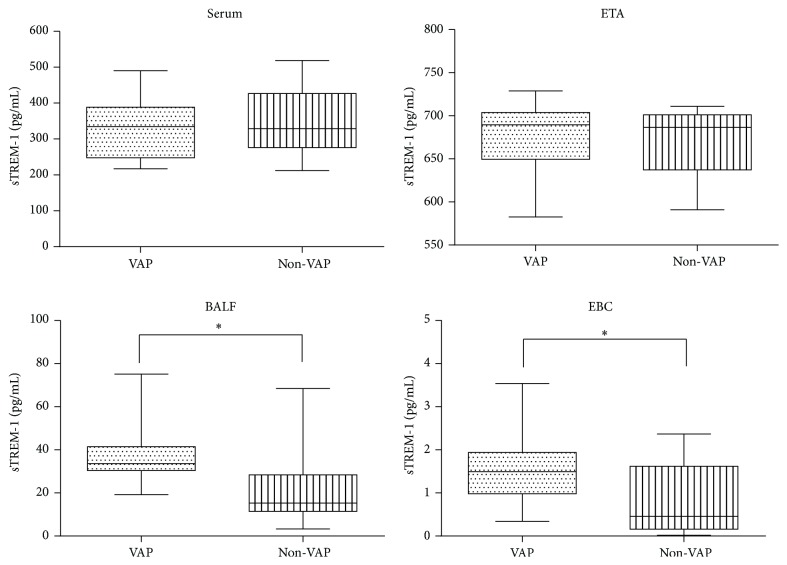
Median soluble triggering receptor expressed on myeloid cells-1 (sTREM-1) levels in serum, endotracheal aspiration (ETA), bronchoalveolar lavage fluid (BALF), and exhaled breath condensate (EBC) samples in 76 patients with and 56 patients without ventilator-associated pneumonia (VAP). In the data bars, the mid-lines represent medians; the tops and bottoms of the bars represent the 25th and 75th percentiles; ^*∗*^*p* < 0.05.

Differences of sTREM-1 levels in serum and ETA between two groups were not statistically significant (*p* > 0.05) (Figure 1).

There were 56 patients who were not VAP among the 132 patients included in our study. In these 56 patients, the average time of suspected VAP was about 76 h. We then also included another 56 patients who needed invasive mechanical ventilation during our study period to be a negative control group. None of the 56 patients in the control group was with infectious diseases. We compared the sTREM-1 levels in 76 h of our 56 non-VAP patients with the negative control group and found that the sTREM-1 concentrations in serum [322.94 (IQR, 276.62–427.72) versus 308.69 (284.77–418.93)] pg/mL, BALF [18.92 (IQR, 11.89–31.72) versus 17.98 (10.92–33.60)] pg/mL, ETA [680.53 (IQR, 642.87–718.72) versus 673.98 (657.82–715.21)] pg/mL, and EBC [0.41 (IQR, 0.19–1.61) versus 0.46 (0.17–1.73)] pg/mL were similar. The results proved that the 56 patients in our study did not have any infectious disease during our study period indeed.

### 3.4. sTREM-1 Sensitivity and Specificity and Diagnostic Values for VAP

**Figure 2:**
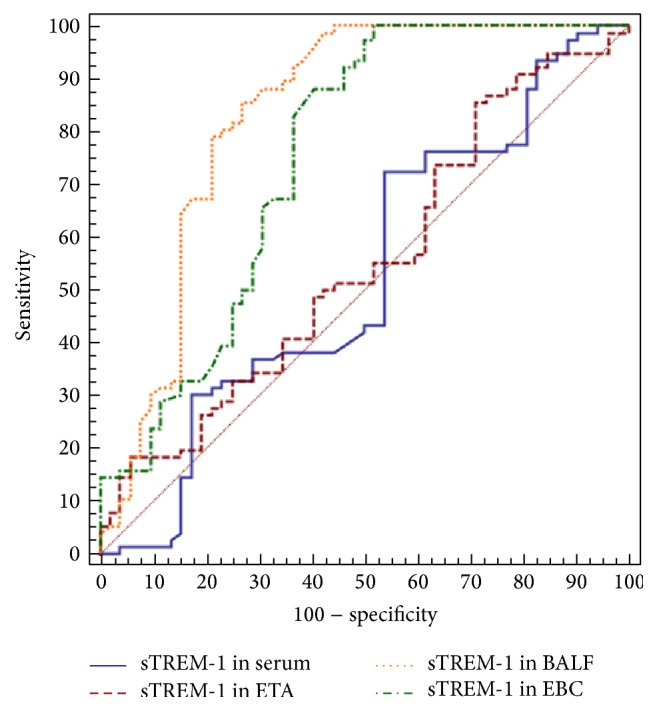
Receiver-operating characteristic curves (ROC) of soluble triggering receptor expressed on myeloid cells-1 (sTREM-1) in serum, endotracheal aspiration (ETA), bronchoalveolar lavage fluid (BALF), and exhaled breath condensate (EBC) samples.

Receiver-operating characteristic curve (ROC) was shown in Figure 2. A cut-off value of 23.61 pg/mL for sTREM-1 in BALF resulted in a sensitivity of 85.5% and specificity of 73.1% which corresponded to the area of 0.831 under the ROC. A cut-off value of 0.31 pg/mL for sTREM-1 in EBC resulted in a relative high sensitivity of 98.3% and low specificity of 48.1% which corresponded to the area of 0.752 under the ROC. The area under the ROC of serum sTREM-1 concentrations was 0.528 and 0.551 of ETA, which were both having low sensitivity and specificity (Table 2).

### 3.5. Correlation of sTREM-1 in Different Samples

**Figure 3:**
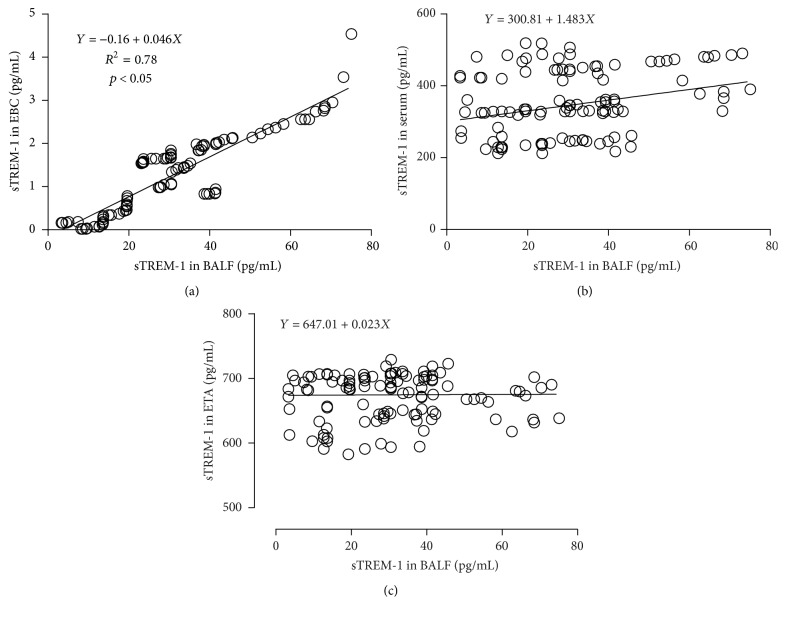
Correlation of sTREM-1 in different samples. (a) Correlation of BALF and EBC sTREM-1 concentration. (b) Correlation of BALF and serum sTREM-1 concentration. (c) Correlation of BALF and ETA sTREM-1 concentration.

A scatter plot was employed to determine whether there were correlations of sTREM-1 between different samples. The results showed that there was a significant correlation between BALF and EBC sTREM-1 levels (*R*^2^ = 0.78, *Y* = −0.16 + 0.046*X*, *p* < 0.05). No statistically significant correlation was identified between the ranks of BALF and serum sTREM-1 values (*Y* = 300.81 + 1.483*X*) or the ranks of BALF and ETA sTREM-1 values (*Y* = 647.01 + 0.023*X*) , *p* > 0.05, Figure 3.

## 4. Discussion

### 4.1. Key Findings

In this prospective multicenter study, all the 132 patients with acute ischemic stroke and suspected VAP underwent bronchoscopy detection. Samples of serum, BALF, ETA, and EBC of these patients were collected and analyzed. We found that the concentration of sTREM-1 in BALF and EBC could effectively categorize patients as VAP positive or VAP negative when using direct bronchoscopic quantitative culture samples as the comparison standard and they had good correlation (*R*^2^ = 0.78). To the best of our knowledge, this is the first study which simultaneously detected concentration of sTREM-1 in serum, ETA, BALF, and EBC in patients who were suspected of VAP.

### 4.2. Relationship to Previous Studies

Despite the major advances that have been achieved in prevention and diagnosis of VAP in the last few years, there are still a large number of unanswered questions. The true incidences of VAP in high risk patients, especially those with ischemic stroke in the ICU, are yet to be determined.

What is more, the diagnostic value of sTREM-1 as a biomarker in determining VAP was controversial. A recent study confirmed that sTREM-1 levels did have diagnostic and prognostic values in VAP [12]. However, other studies reported poor sensitivity and specificity of sTREM-1 to detect patients with pneumonia [13, 14]. An important explanation for the disparity lies in the difference in study populations. Some studies did not exclude patients who were immunocompromised which might lead to false negative results of sTREM-1 assay. Others did not exclude the patients who had extrapulmonary infection during ICU stay which increased the sTREM-1 concentration that was not the result of VAP. The value of BALF sTREM-1 in pneumonia was also controversial because of the noninfective influence factors, such as tuberculosis and connective tissue diseases [15]. Additionally, an earlier study indicated that it was hard to classify patients as VAP positive or VAP negative by using CPIS and sTREM-1 [16].

Procalcitonin (PCT) is a prohormone secreted into serum most likely from neuroendocrine cells in the lungs or intestine as part of the systemic inflammatory response. The rapid release and long half-life of procalcitonin make it potentially useful as a diagnostic indicator of VAP. Four studies report the use of serum PCT as a biomarker in diagnosing VAP [17–20]. These studies revealed that sensitivities ranged between 41 and 100% with lower sensitivities indicating the potential to miss many positive VAP patients. Specificity was higher in two of four studies with a range of 97–100%. Variable cut-off values and dissimilar study designs across the studies contribute to the difficulty in interpreting the results. The different patient populations across studies may have contributed to elevated PCT levels that were unrelated to VAP. The most important thing is that some interference factors like previous antibiotics use did affect the mean serum PCT level and sensitivity and specificity which suggested that serum PCT was not a good biomarker for VAP [21].

Therefore, new biomarkers are urgently needed to diagnose VAP especially for patients with ischemic stroke due to its high mortality. So we detected the concentrations of sTREM-1 and expected that it had higher sensitivity and specificity than PCT.

### 4.3. Study Significance

Biomarkers can be detected in any biological sample including serum, ETA, BALF, and EBC. A biomarker for VAP should be low or absent when infection is not present and elevated in the presence of infection. sTREM-1 belongs to the immunoglobulin superfamily, which is expressed in neutrophils, monocytes, and macrophages in the course of acute inflammatory response. sTREM-1 triggers the secretion of proinflammatory mediators through a signaling pathway (DAP12) and functions as an amplifier of the inflammatory response. This characteristic leads to its high sensitivity and poor specificity in infectious diseases. In response to infection, sTREM-1 is either secreted or shed and can be measured in body fluids and is almost undetectable in patients with nonmicrobial inflammation. So in patients with pneumonia, it is a better choice to detect the concentration of sTREM-1 in BALF than in serum which leads to a relative higher specificity.

One previous study reported that sTREM-1 increased in serum samples of patients with pneumonia and in EBC samples of patients with VAP [15]. Moreover, the raise of sTREM-1 level in pleural effusion, cerebrospinal fluid, urine, and synovial fluid also has important clinical significance in identifying the infectious diseases [22]. Therefore, sTREM-1 as a biomarker for the diagnosis of inflammatory diseases is of high sensitivity and low specificity. According to the characteristics of sTREM-1 expression, it is concluded that analyses of serum sample alone were not able to distinguish between the systemic acute inflammatory response and local infection accurately, although in some patients it could reflect the severity of the infection especially in neonatal sepsis [23]. In our study, the specificity of serum sTREM-1 in diagnosing VAP was only 46.2%. We acquired ETA samples via the artificial airway (endotracheal tube) which could inevitably be contaminated by the bacterial colonization, leading to misinterpretation of the study results. Therefore, our study also analyzed sTREM-1 levels in BALF and EBC in order to achieve more accurate evaluation.

Damage of the tracheal mucous membrane and stress could all lead to the raise of sTREM-1 in serum due to its high sensitivity and low specificity which resulted in the poor performance in VAP diagnosis. The area under the ROC is only 0.528. The incidence of VAP will increase along with the incidence of bacterial colonization when mechanical ventilation was prolonged which will lead to the false positive result of sTREM-1 in ETA. In our study, the specificity of sTREM-1 in ETA was only 28.8%, which could not diagnose VAP accurately.

sTREM-1 as an inflammatory biomarker could be detected in epithelial lining fluid which could also be detected in BALF indirectly that was secreted by lung which was infected as the target organ. Detection of sTREM-1 in BALF was a more effective way to effectively categorize patients as VAP positive or VAP negative and it was less intrusive to extrapulmonary infection. Our study also confirmed that on the day of clinically suspected VAP sTREM-1 in BALF had high sensitivity and specificity with 0.831 area under the ROC which indicated that it had higher diagnostic accuracy of VAP. An increasing number of researches on EBC in the diseases of chronic obstructive pulmonary disease, asthma, and other airway inflammatory diseases were published [24–26]. However, studies on EBC in pneumonia or VAP were relatively rare. What is more, the results of these studies were still controversial because the methods of EBC acquisition, preservation, and detection have not been standardized.

EBC fluid was collected in a plastic container located at the center of the exhaled portion of the ventilator tubing that was far away from the artificial airway. Although the level of EBC was relatively low because it was composed of expiratory air, it had a high sensitivity (98.3%) in diagnosing VAP. However, detection and analysis of EBC were inevitably affected by the bacterial colonization in the artificial airway, which resulted in an unsatisfactory specificity (48.1%). At all events, the area under ROC of sTREM-1 in EBC could reach 0.752, and the concentration of sTREM-1 in EBC had good correlation with sTREM-1 in BALF (*R*^2^ = 0.78), indicating that it might be one of the accessory biomarkers in diagnosing VAP. As the collection of EBC is noninvasive, it is promising in clinical practice in the future with the standardized methods.

This study has several limitations. We included a homogeneous population of ischemic stroke which might minimize the heterogeneity and avoided misinterpreting the study result. However, its application to routine clinical practice remains uncertain. Whether the detection of BALF sTREM-1 in general may translate into clinical benefits outside this setting deserves further explorations.

## 5. Conclusion

In conclusion, sTREM-1 levels in BALF and EBC accurately distinguished patients of acute ischemic stroke with or without VAP when using direct bronchoscopic quantitative culture samples as the reference standard. We can expect the routine use of sTREM-1 levels in BALF or EBC to diagnose patients with VAP in the future with the standardization of samples improvement.

## Figures and Tables

**Table 1:** Characteristics of the study groups (mean ± SD/%/IQR).

Characteristics	All patients (*n* = 132)	Patients with VAP (*n* = 76)	Patients without VAP (*n* = 56)	*p*
Age, yrs	70.27 ± 11.43	69.39 ± 10.84	70.89 ± 11.36	0.447
Male gender, *n* (%)	63 (47.7)	37 (48.7)	26 (46.4)	0.861
Previous medical illness				
Cardiovascular disease, *n* (%)	70 (53.03)	39 (51.31)	31 (55.35)	0.725
Cerebrovascular disease, *n* (%)	41 (31.06)	23 (30.26)	18 (32.14)	0.851
Position of stroke				
TACI, *n* (%)	48 (36.36)	29 (38.16)	19 (33.93)	0.715
POCI, *n* (%)	84 (63.64)	47 (61.84)	37 (66.07)	0.715
Smoking history	39 (29.55)	24 (31.58)	15 (26.78)	0.571
Hypoproteinemia	32 (24.24)	17 (22.37)	15 (26.79)	0.682
APACHE II	17.92 ± 4.94	18.21 ± 5.48	17.52 ± 4.88	0.442
GCS	5.71 ± 2.27	5.82 ± 2.35	5.69 ± 2.78	0.085
mCPIS	3.42 ± 1.45	3.37 ± 1.28	3.45 ± 1.61	0.751
PO_2_/FiO_2_ (mmHg)	112.67 ± 50.11	115.75 ± 48.58	108.92 ± 52.78	0.443

TACI, total anterior circulation infarcts; POCI, posterior circulation infarcts; APACHE II, acute physiology and chronic health evaluation; GCS, Glasgow Coma Scale; mCPIS, modified clinical pulmonary infection score.

**Table 2:** Best cut-off values of sTREM-1 that were obtained from ROC curves for VAP diagnosis.

Variables	AUC	95% CI	Cut-off (pg/mL)	Sensitivity%	Specificity%
sTREM-1 in serum	0.528	0.438*～*0.617	358.93	72.4	46.2
sTREM-1 in ETA	0.551	0.461*～*0.638	641.94	85.5	28.8
sTREM-1 in BALF	0.831	0.755*～*0.892	23.61	85.5	73.1
sTREM-1 in EBC	0.752	0.668*～*0.824	0.32	100	48.1

AUC = area under the curve.

## Abbreviations

APACHE II: Acute physiology and chronic health evaluation
BAL: Bronchoalveolar lavage
BALF: Bronchoalveolar lavage fluid
CFU: Colony-forming units
CPIS: Clinical pulmonary infection score
EBC: Exhaled breath condensate
ELISA: Enzyme-linked immunosorbent assay
ETA: Endotracheal aspiration
GCS: Glasgow Coma Scale
ICU: Intensive care unit
IQR: Interquartile range
PCT: Procalcitonin
POCI: Posterior circulation infarcts
ROC: Receiver-operating characteristic curves
sTREM-1: Soluble triggering receptor expressed on myeloid cells-1
TACI: Total anterior circulation infarcts
VAP: Ventilator acquired pneumonia.
